# Macrophage Trem2 deficiency aggravates aging-induced vascular remodeling by acting as a non-classical receptor of interleukin-13

**DOI:** 10.1186/s43556-025-00377-1

**Published:** 2025-12-29

**Authors:** Youming Chen, Zhaoxiang Zeng, Zetao Wei, Yi Zhan, Luling Wu, Xinlin Zhu, Meifang Li

**Affiliations:** 1 Department of Infectious Diseases and Immunology, Shanghai Public Health Clinical Center, Fudan University, Shanghai, 201508 China; 2 Department of Vascular Surgery, Shanghai General Hospital, Shanghai Jiaotong University, Shanghai, 201620 China; 3 Department of Emergency, Dan Zhou People’s Hospital, Danzhou, Hainan 271706 China; 4 Department of Radiology, Shanghai Public Health Clinical Center, Fudan University, Shanghai, 201508 China; 5 Department of Endoscopy, School of Medicine, Shanghai Pulmonary Hospital, Tongji University, Shanghai, 200082 China; 6 Department of Dermatology, Shanghai Key Laboratory of Medical Mycology, Shanghai Changzheng Hospital, Naval Medical University, Shanghai, 200003 China; 7 The Center for Fungal Infectious Diseases Basic Research and Innovation of Medicine and Pharmacy, Ministry of Education, Shanghai, 200433 China; 8 Department of Emergency, Shanghai Sixth People’s Hospital Affiliated to Shanghai Jiao Tong University School of Medicine, Shanghai, 200233 China; 9 Department of Emergency, Jinjiang Municipal Hospital (Shanghai Sixth People’s Hospital Fujian), Jinjiang, Fujian 362200 China

**Keywords:** Vascular aging, Trem2, Macrophage, IL-13, Metabolic reprogramming, α-KG

## Abstract

The receptor for triggering expressed on myeloid cells 2 (Trem2), which is a key hub of immune signals, is a cell-surface receptor expressed selectively in myeloid cells. Macrophages have multi-faceted functions in vascular aging. However, the function of Trem2 and its ligands in vascular aging has not been described. Here, we investigated Trem2’s function in aging vasculature using transcriptome analysis, western blotting, and quantitative polymerase chain reaction (qPCR) to assess its expression. Aged (24-month-old) wild-type mice exhibited significantly upregulated Trem2 in aortic senescent macrophages compared to young (2-month-old) controls. Compared with littermate controls, aged mice with macrophage-specific Trem2 knockout (T2-cKO) developed exacerbated arterial stiffness, impaired vascular contractility, and an acceleration of histological aging markers. Trem2 deficiency intensified aortic inflammatory responses and oxidative stress. Mechanistically, interleukin (IL)-13 from senescent macrophages directly bound Trem2, activating the Syk-Sp1-SLC25A51 pathway to enhance mitochondrial nicotinamide adenine dinucleotide (NAD)⁺ transport. This triggered metabolic reprogramming, increasing alpha-ketoglutarate (α-KG) production, which modulated vascular smooth muscle cell (VSMC) phenotype. Notably, α-KG supplementation in vivo rescued Trem2 deficiency-driven vascular aging and dysfunction. Our study identifies the IL-13/Trem2 axis as a protective mechanism against vascular aging via α-KG-dependent metabolic crosstalk between macrophages and VSMCs. Thus, Trem2 may be a treatment target for diseases related to vascular aging.

**Supplementary Information:**

The online version contains supplementary material available at 10.1186/s43556-025-00377-1.

## Introduction

Vascular aging is a key factor of risk for cardiovascular disease onset (CVDs); CVDs have become quite prevalent and have emerged as the principal cause of death globally [1, 2]. As individuals age, blood vessels undergo pathological remodeling and lose their integrity and functionality, resulting in aging-related CVDs [1, 3, 4]. However, owing to the prominent heterogeneity of vascular walls consisting of cells from various origins, the molecular features and mechanisms of human blood vessels that are aged remain obscure. Therefore, elucidating the mechanisms underlying vascular aging is vital for preventing and treating CVDs.

In the process of vascular aging, macrophages undergo senescence in response to alterations in the microenvironment and changes in various vascular stimuli, such as oxidative stress and DNA damage [5–7]. Moreover, these aging macrophages can drive vascular pathological remodeling through multiple mechanisms, such as inflammation, dysregulation of lipid metabolism, and impairment of tissue repair. Studies on immunometabolism have recently revealed that changes in the macrophage metabolic phenotype establish their activation states and functions, which has attracted extensive attention to the metabolic reprogramming of macrophages [8–11]. However, the metabolism and regulation of senescent macrophages, as well as the underlying mechanisms of the crosstalk between cells, in vascular aging remain unclear.

Receptor of triggering expressed on myeloid cells 2 (Trem2) is a transmembrane receptor of the immunoglobulin family expressed mainly on brain microglia and peripheral macrophages [12, 13]. Owing to its ability to bind to various ligands, the function of Trem2 has attracted extensive research interest, with a focus on central neurodegenerative diseases, especially Alzheimer’s disease (AD) [12–18]. Trem2 serves as an important controller of crucial cellular functions in the central nervous system, which include proliferation, metabolism, phagocytosis, and chemotaxis, by detecting various signal ligands, such as lipoproteins, phospholipids, and bacterial products [12–15]. In the periphery, Trem2 also participates in the occurrence and progression of diabetes, liver-related disease, and atherosclerosis by regulating lipid metabolism, inflammation signaling, the viability of immune cells, and apoptosis [16–18]. Lipid metabolism and inflammation play important roles in vascular aging. Nevertheless, the exact function of Trem2 in this context continues to be obscure.

In this investigation, we showed that Trem2 is key in vascular aging. Macrophage Trem2 deficiency aggravates aging-induced vascular reshaping and dysfunction. Comprehensive mechanistic studies on senescent macrophages revealed that interleukin (IL)−13 binds directly to Trem2, which promotes mitochondrial nicotinamide adenine dinucleotide (NAD)^+^ transport and regulates the vascular smooth muscle cell (VSMC) phenotype by the metabolic reprogramming of alpha-ketoglutarate (α-KG) via a paracrine mechanism. These results indicated that the interaction between macrophage IL-13 and Trem2 is important in vascular aging.

## Results

### Increased expression of Trem2 in macrophages in the aortas of aged mice

**Fig. 1:**
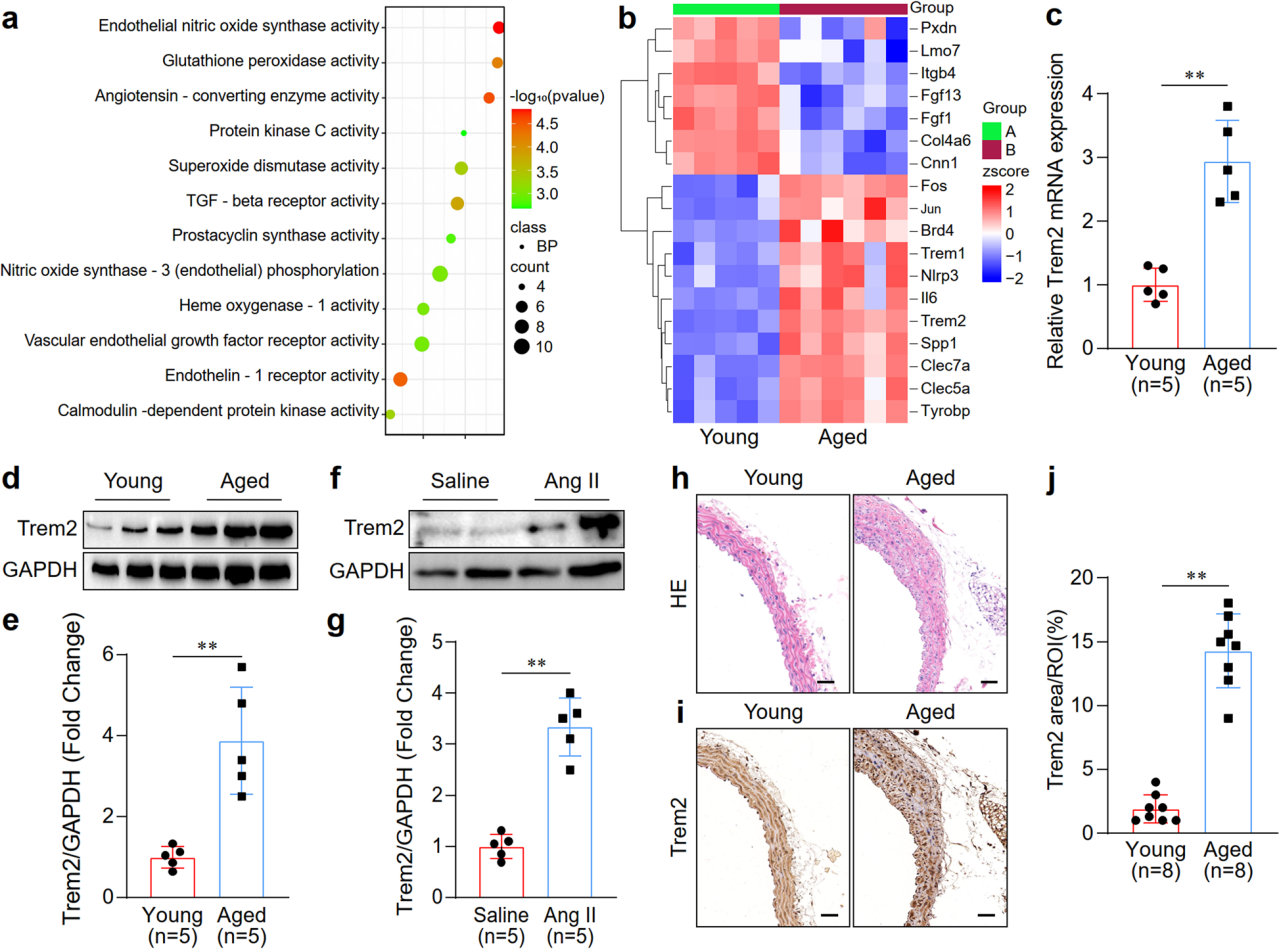
Trem2 levels were increased in macrophages of the aortas of aged mice. **a** Gene ontology analysis of the differentially expressed genes (DEGs) in aorta tissues between young and aged mice. **b** Heatmap of gene expression patterns in young and aged WT mouse aortas. **c** Trem2 expression was detected in aorta samples of young and old WT mice via qPCR. *n* = 5 samples/group. **d** Western blotting analysis of Trem2 levels in aorta samples of young and aged WT mice. *n* = 5 samples per group. **e** Quantified data of the band intensity of immunoblotting in d. **f** Western blotting analysis of Trem2 levels in aorta samples of Ang II-induced and control mice. *n* = 5 samples/group. **g** Quantified data the band intensity of immunoblotting in f. **h** H&E staining analysis in aorta samples of young and aged WT mice. Scale bar: 100 µm. *n* = 8 samples per group. **i** Trem2 expression was analysed by immunohistochemical staining in aorta samples of young and aged WT mice. Scale bar is 100 µm. *n* = 8 samples/group. **j** Quantified data of immunohistochemical staining intensity in i. **p < 0.01

To identify genes that may be associated with vascular aging, we examined and compared the profiles of gene expression of the aortic tissues of old and young wild-type (WT) mice via RNA sequencing (RNA-seq). The analysis results of gene ontology revealed that the differentially expressed genes (DEGs) in the mouse aorta tissues were related to aging, vascular remodeling, and oxidative stress (Fig. 1a). Trem2 was upregulated in the aortic tissues of aged mice (Fig. 1b). This finding was verified via quantitative polymerase chain reaction (qPCR) and western blotting analyses of mouse aortic samples (Fig. 1c-e). The cells related to Trem2 expression were identified via flow sorting in old and young mice. The results of the qPCR assays revealed that Trem2 was expressed only in macrophages, with a significantly greater level of expression in the aged group (Fig. S1a). Additionally, angiotensin II (Ang II) induces vascular remodeling as part of vascular aging [19]. Thus, we evaluated the expression of Trem2 in the Ang II-treated and control mouse aortas and found that Trem2 expression was elevated in the aortas of Ang II-treated mice compared to that in the aortas of saline-treated control mice (Fig. 1f, g). The thickness of the middle artery and the ratio of the middle artery area to the vascular lumen were markedly greater in aged mice than in young mice (Fig. 1h and S1b-c). Furthermore, immunohistochemical analysis of aortic sections from mice demonstrated an elevated expression of Trem2 in the vasculature of aged mice relative to their young counterparts (Fig. 1i, j). These results indicated that the levels of Trem2 protein in macrophages were higher in the aged mouse aortas.

### Macrophage-specific deletion of Trem2 accelerates vascular aging and dysfunction

**Fig. 2:**
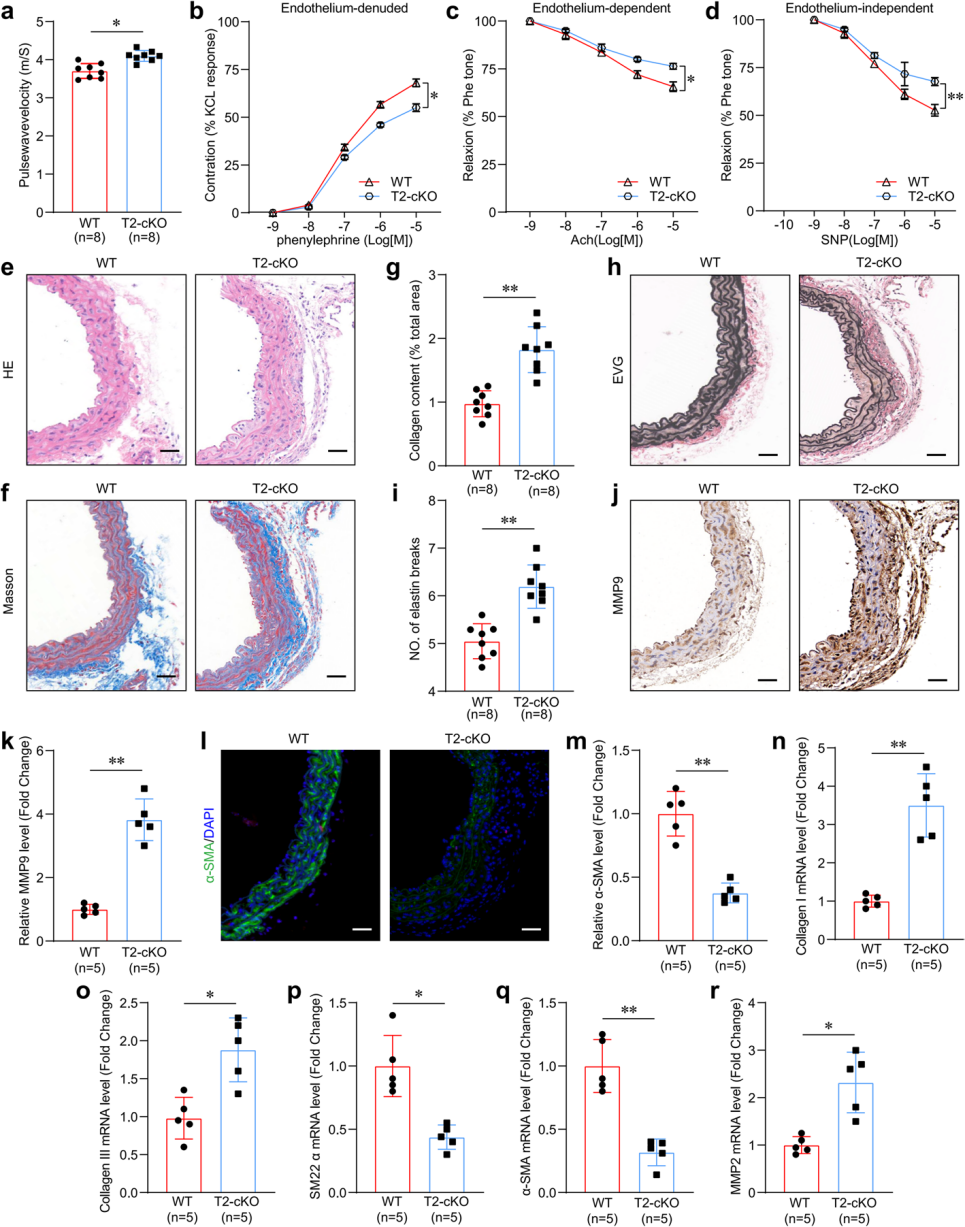
Macrophage Trem2 deficiency accelerated vascular aging and dysfunction. **a** PWV measurements in aged WT and T2-cKO mice. *n* = 8 samples/group. **b-d** Ex vivo analysis of the vascular constriction-relaxation function of aortas between aged WT and aged T2-cKO mice. *n* = 8 samples/group. **b** Phenylephrine-mediated arterial vessel contractions. **c** Relaxation dependent on the endothelium in response to Ach. **d** Relaxation independent of the endothelium in response to nitric oxide donor sodium nitroprusside (NO-donor SNP), an agonist independent of the endothelium. **e** H&E staining analysis in aorta samples of aged WT and aged T2-cKO mice. Scale bar: 100 µm. *n* = 8 samples per group. **f, g** Images and quantification of Masson staining of aorta samples of aged WT and aged T2-cKO mice. Scale bar: 100 µm. *n* = 8 samples per group. **h, i** Images of Elastin van Gieson (EVG) staining of elastin fibers in aorta samples of aged WT and T2-cKO mice. Scale bar is 100 µm. *n* = 8 samples/group. **j, k** Images of immunohistochemical staining of MMP9 and quantification in aorta samples of aged WT and aged T2-cKO mice. Scale bar: 100 µm. *n* = 5 samples per group. **l, m** Immunofluorescence staining images of α-SMA and quantification in aorta samples of aged WT and aged T2-cKO mice. Scale bar is 100 µm. *n* = 5 samples/group. **n-r** The expressions of Collagen I, Collagen III, SM22α, α-SMA and MMP-2 in aorta samples of aged WT and T2-cKO mice. *n* = 5 samples per group. *p < 0.05, **p < 0.01

The increased expression of Trem2 in the macrophages of aged aortas prompted our investigation of the role of Trem2 in vascular dysfunction induced by age. Male macrophage-specific Trem2 knockout (T2-cKO) mice together with their WT littermates were kept for 24 months to assess the function of macrophage Trem2 expression in vascular aging. The macrophage Trem2 effects on vascular function were evaluated by measuring the pulse wave velocity (PWV), which serves as the gold standard of arterial stiffness and vascular aging [20]. Compared to their WT littermates, aged T2-cKO mice presented considerably higher PWV values (Fig. 2a). To investigate the influence of macrophage Trem2 on arterial constriction-relaxation function, the aortas were dissociated to conduct an ex vivo functional study. Compared to old WT mice, old T2-cKO mice displayed less vascular constriction induced by phenylephrine (PE) in endothelium-denuded aortas and poorer relaxation induced by the endothelium-reliant relaxant acetylcholine (Ach) and the endothelium-independent relaxant sodium nitroprusside (SNP) (Fig. 2b-d). These findings indicated that Trem2 is involved in aging-induced vascular remodeling and that macrophage Trem2 loss exacerbates aging-induced vascular dysfunction.

Histological analyses were performed to determine whether the exacerbation of vascular dysfunction in aged T2-cKO mice was coupled with vascular remodeling. Hematoxylin and eosin (H&E) staining revealed that compared to those in old WT mice, the thickness of the middle artery and the ratio of the area of the middle artery to the vascular lumen in aged T2-cKO mice were significantly greater (Fig. 2e and S1b-c). T2-cKO exacerbated other vascular aging characteristics, such as collagen deposition (Fig. 2f, g) and the breakage of elastin fibers (Fig. 2h, i). Matrix metalloproteinases (MMPs) are required for vascular aging [21]. Compared to those of their littermate control mice, the aortas of aged T2-cKO mice presented greater expression of MMP9 (Fig. 2j, k). Given that the alteration in the contractile phenotype of VSMCs is another significant feature of vascular aging [22], we also found that the aortas of aged T2-cKO mice were associated with a decrease in vascular contraction function characterized by the downregulation of α-SMA and SM22α (Fig. 2l, m). To further validate these findings at the transcriptional level, we performed qPCR analysis. The results demonstrated a consistent upregulation of Collagen I, Collagen III, and Mmp2 mRNA, concomitant with a marked downregulation of the contractile phenotype markers SM22α and α-SMA in the aortas of aged T2-cKO mice compared to aged WT controls (Fig. 2n-r). These findings suggested that macrophage Trem2 loss may regulate the phenotype of VSMCs to contribute to vascular remodeling and stiffness dependent on age.

### Macrophage-specific loss of Trem2 reprograms the vascular transcriptome and exacerbates vascular inflammation, oxidative stress, and apoptosis

**Fig. 3:**
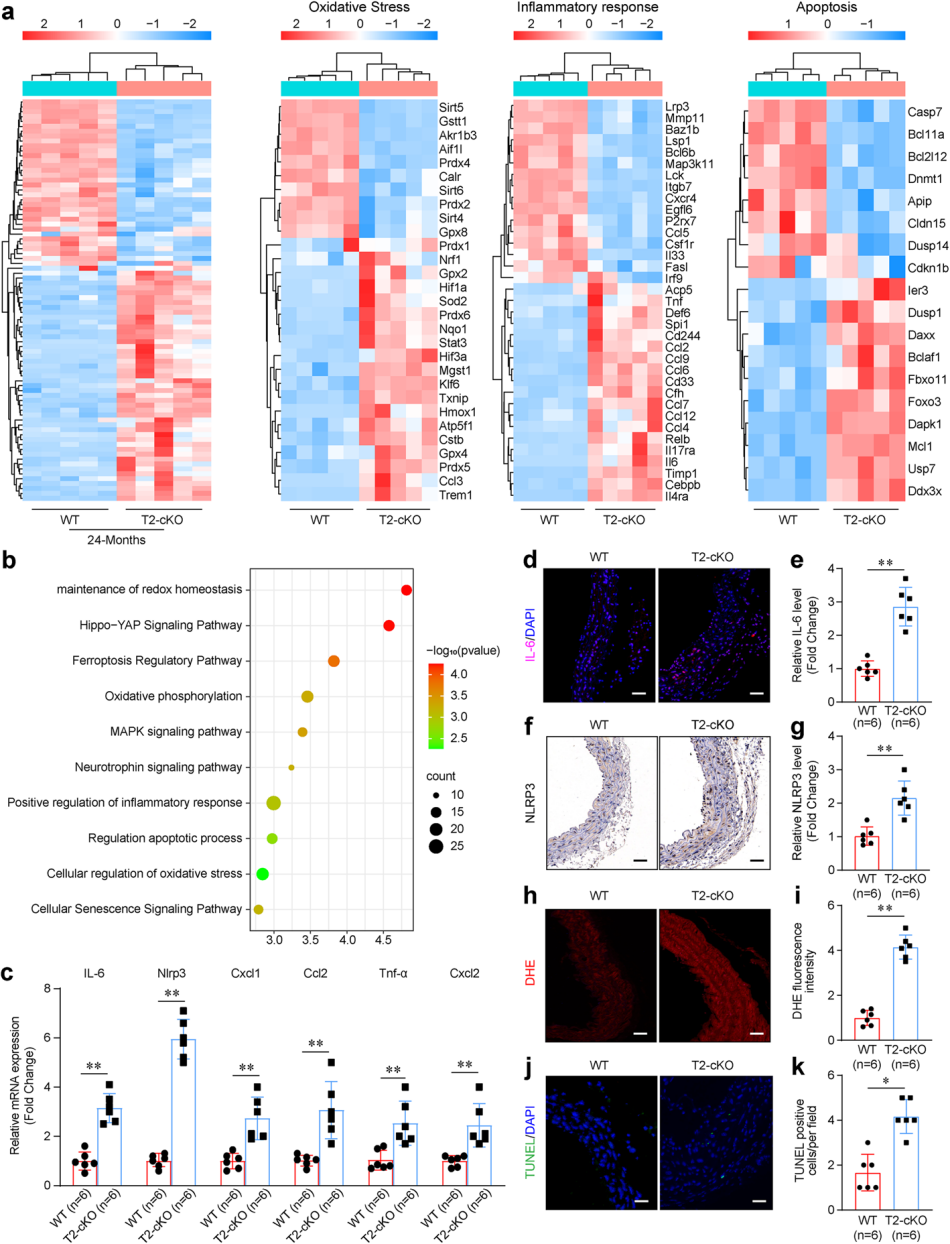
Macrophage Trem2 loss regulates gene signatures associated with inflammatory responses and oxidative stress in vascular aging. **a-b** RNA-seq analysis of the transcriptome of aged aortas of T2-cKO mice and their littermates. *n* = 5 samples/group. Gene ontology and KEGG pathway analysis of the DEGs in aged WT and T2-cKO mouse aortas. **c** QPCR quantification of the gene expressions of the inflammatory responses in aged aortas of WT and T2-cKO mice. *n* = 6 samples per group. **d, e** Immunohistochemical staining images of IL-6 and quantification in aged aortas of WT and T2-cKO mice. Scale bar: 100 µm. *n* = 6 samples per group. **f, g** Immunohistochemical staining images of NLRP3 and quantification in aged aortas of WT and T2-cKO mice. Scale bar is 100 µm. *n* = 6 samples/group. **h, i** Images of DHE staining and quantification in aged aortas of WT and T2-cKO mice. Scale bar: 100 µm. *n* = 6 samples per group. **j, k** TUNEL staining images and quantification in aged aortas of WT and T2-cKO mice. Scale bar: 100 µm. *n* = 6 samples per group. *p < 0.05, **p < 0.01

To clarify the mechanisms underlying the function of macrophage Trem2 in vascular aging, RNA-seq was conducted to analyze the DEGs of aortas from old WT and T2-cKO mice. Transcriptome analysis revealed that macrophage Trem2 deficiency enriched the gene sets involved in inflammation, oxidative stress, apoptosis, and immune regulation (Fig. 3a, b). To validate these findings, we determined the expression of genes related to inflammatory responses by conducting qPCR and found that the mRNA levels of IL-6, NLRP3, TNF-a, CXCL1, CXCL2, and CCL2 in the aorta were higher in aged T2-cKO mice than in aged control mice (Fig. 3c). Immunofluorescence staining and histochemical staining revealed that macrophage Trem2 knockout promoted aging-induced overexpression of IL-6 and NLRP3 in the mouse aortas (Fig. 3d-g). Furthermore, we investigated the effects of macrophage Trem2 on oxidative stress and apoptosis. The loss of macrophage Trem2 increased the total superoxide level and the apoptotic cell numbers in the aortas of aging-induced mice (Fig. 3h-k). These findings indicated that macrophage Trem2 significantly enhances inflammatory responses and oxidative stress during vascular aging.

### Trem2 is a non-classical receptor of IL-13 in senescent macrophages

**Fig. 4:**
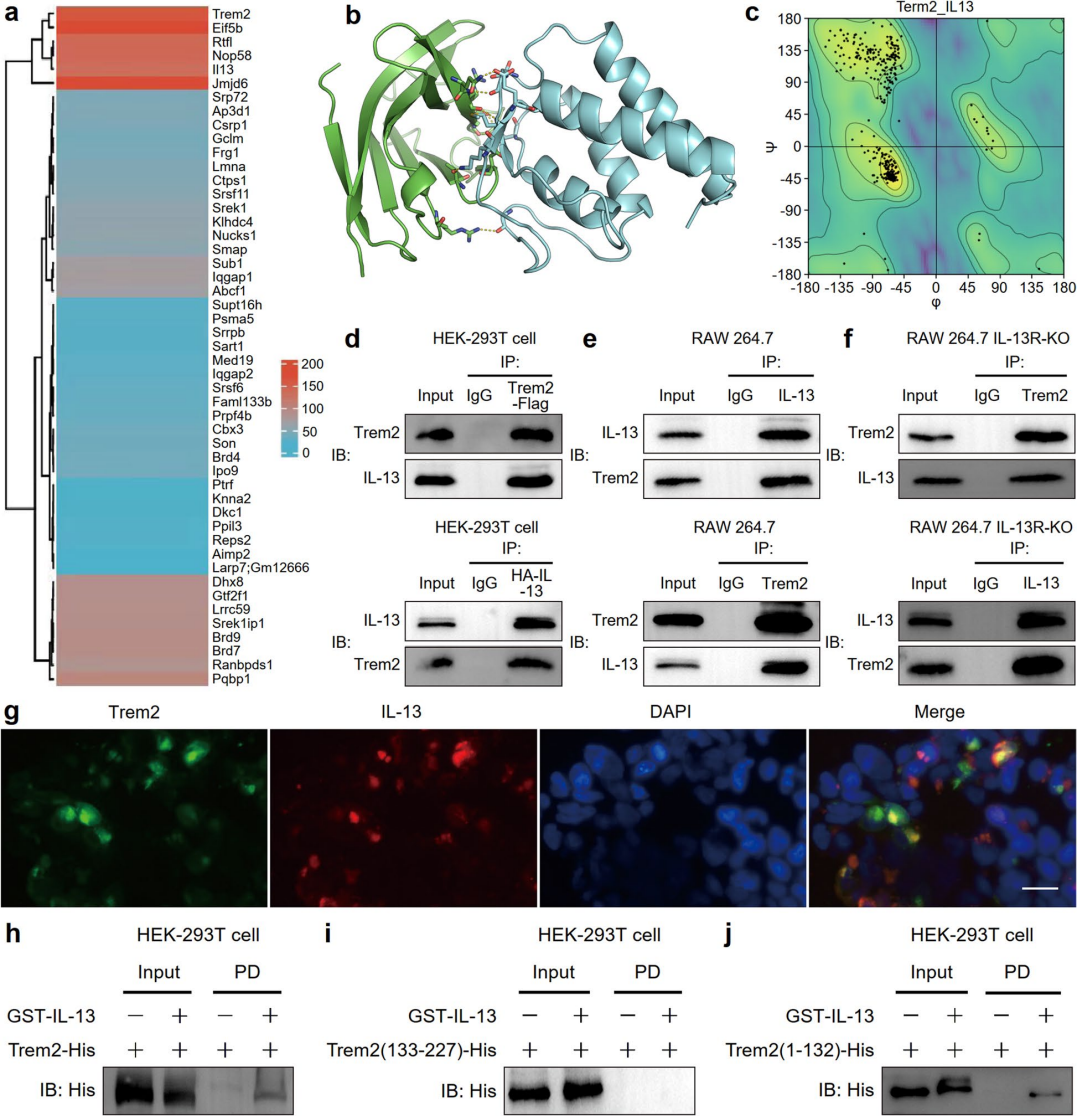
Trem2 is a non-classical receptor of IL-13 in macrophages of vascular aging. **a** Heatmap of Trem2 top 50 target proteins in macrophage senescence induced by Ang II. **b** Computational modeling of the three-dimensional complex structure of IL-13 and Trem2 using the Alphafold-multitimer Algorithm. **c** Ramachandran plot analyzing the conformational plausibility of the IL-13/Trem2 binding. **d** Trem2 and IL-13 co-immunoprecipitation in HEK-293 T cells. **e, f** Endogenous Trem2 and IL-13 co-immunoprecipitation in RAW 264.7 and RAW 264.7 IL-13-KO. **g** Immunofluorescence analysis of Trem2 co-localization with IL-13 on the macrophages. Scale bar: 100 µm. **h** GST pull-down assay of GST-IL-13 binding directly to His-Trem2. **i, j** GST pull-down assay of the interactions between GST-fused IL-13 and His-Trem2 protein fragments

Trem2 is an immunologically significant receptor protein. It orchestrates various biological phenomena through ligand-receptor interactions [12–15]. Thus, we searched for ligands of Trem2 in senescent macrophages via mass spectrometry. Bone marrow-derived macrophages (BMDMs) were harvested from WT mice and then induced to differentiate into macrophages. Mass spectrometry was conducted after macrophage transfection with the Trem2-overexpressing lentivirus and treatment with Ang II for 72 h to simulate an aging phenotype in vitro (Fig. S2). The top 50 proteins with the highest scores are displayed in Fig. 4a, and IL-13 was unexpectedly incorporated. To confirm whether IL-13 can serve as a ligand for Trem2, we first used the AlphaFold multimer to forecast the complex IL-13 and Trem2 structure (Fig. 4b) and then constructed a Ramachandran plot to investigate the dihedral angle distribution of individual residues from the predicted protein structures (Fig. 4c). Coprecipitation assays were conducted to validate the interaction between IL-13 and Trem2. HEK293T cells were transfected with either Trem2 or IL-13, and the results showed that IL-13 could interact with Trem2 in these cells (Fig. 4d). Consistent findings were obtained in RAW 264.7 cells (Fig. 4e) and RAW 264.7 cells with IL-13 receptor knockout (IL-13R-KO) (Fig. 4f). These results confirmed that IL-13 binds Trem2 independently of IL-13R. Immunofluorescence examination revealed that Trem2 colocalized with IL-13 on macrophages, providing visual evidence for their physical interaction (Fig. 4g). Next, plasmids expressing the full-length sequences of mouse Trem2 or IL-13 were constructed, and we purified the Trem2 protein using GST-IL-13 and an N-terminal His tag. GST pulldown assays demonstrated that His-Trem2 bound to GST-IL-13 (Fig. 4h). To identify the specific Trem2 region that accounts for the IL-13 interaction, we also constructed N-terminal aa1-aa132 of Trem2 (NT-aa1-132) and NT aa133-227 of Trem2 (NT-aa133-227) tagged with His from two small fragments of Trem2 and expressed them in HEK-293 T cells. The results revealed that GST-tagged IL-13 interacted with the full-length Trem2 and the fragment of NT-aa1-132 (Fig. 4j), but not the fragment of NT-aa133-227 (Fig. 4i). These findings indicated that Trem2 acts as a non-classical receptor for IL-13 in senescent macrophages.

### IL-13/Trem2 in combination with macrophages retards vascular aging by regulating the phenotype of VSMCs in a paracrine manner

**Fig. 5:**
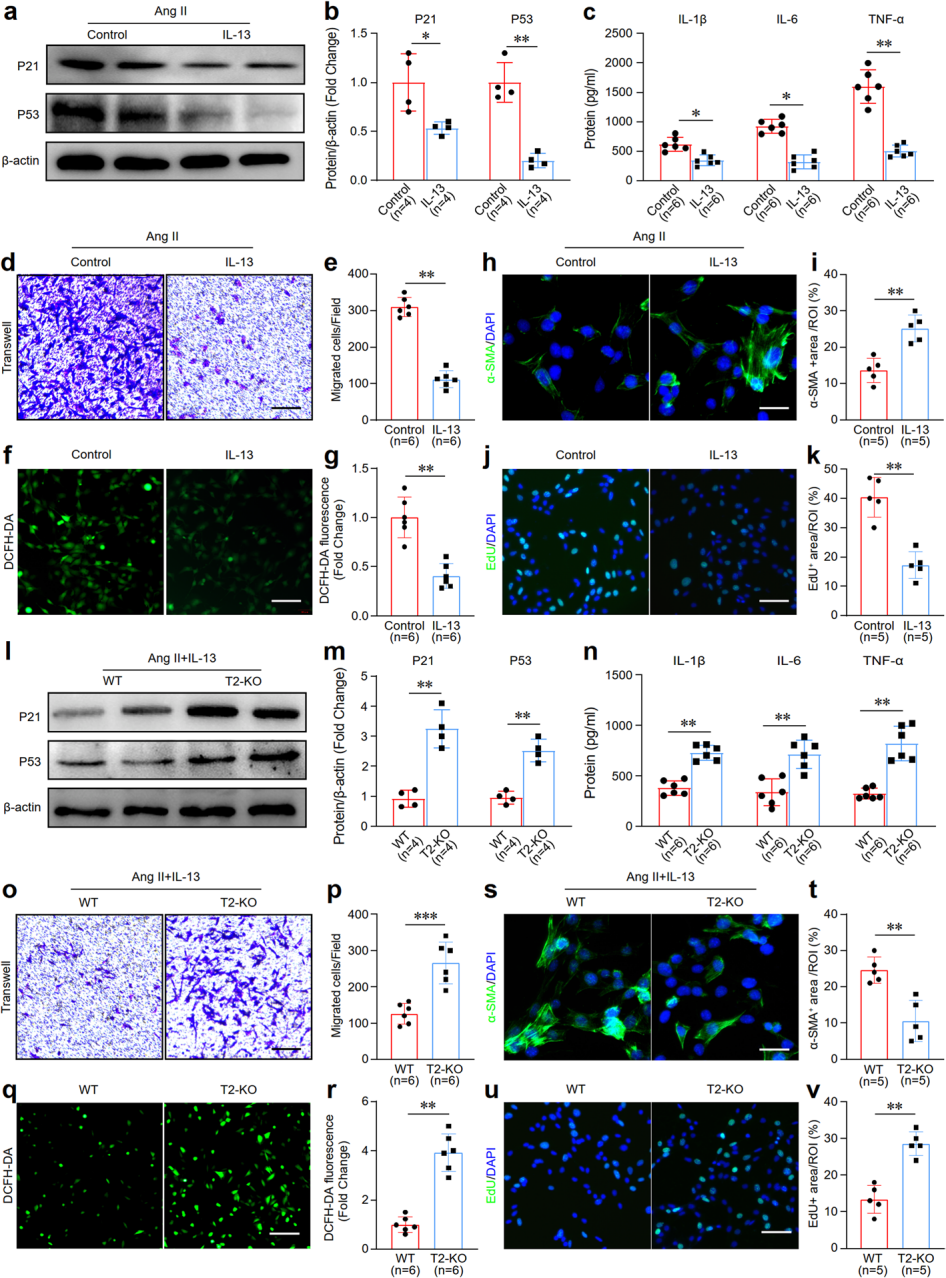
IL-13/Trem2 combination in macrophages retards vascular aging by paracrinely regulating the phenotype of VSMCs in a paracrine manner. **a, b** Western blotting images and quantification of senescence phenotypic marker proteins P21 and P53 in senescent macrophages treated with IL-13. *n* = 4 samples per group. **c** ELISA detected the levels of SASPs in senescent macrophages treated with IL-13. *n* = 6 samples per group. **d, e** Transwell assay images and quantification data of VSMCs co-cultured with IL-13-treated senescent macrophages. *n* = 6 samples/group. **f, g** DCFH-DA staining images and quantification data of VSMCs co-cultured with IL-13-treated senescent macrophages.* n* = 6 samples per group. **h, i** Immunofluorescence staining images and quantification data of α-SMA protein in VSMCs co-cultured with IL-13-treated senescent macrophages. *n* = 5 samples per group. **j, k** Edu/DAPI staining images and quantification data of VSMCs co-cultured with IL-13-treated senescent macrophages. *n* = 5 samples per group. **l, m** Western blotting images and quantification of senescence phenotypic marker proteins P21 and P53 in senescent Trem2 knockout macrophages treated with IL-13. *n* = 4 samples/group. **n** ELISA detected the levels of SASPs in senescent Trem2 knockout macrophages treated with IL-13. *n* = 6 samples per group. **o, p** Transwell assay images and quantification data of VSMCs co-cultured with IL-13-treated senescent Trem2 knockout macrophages. *n* = 6 samples per group. **q, r** DCFH-DA staining images and quantification data of VSMCs co-cultured with IL-13-treated senescent Trem2 knockout macrophages. *n* = 6 samples per group. **s, t** Immunofluorescence staining images and quantification data of α-SMA protein in VSMCs co-cultured with IL-13-treated senescent Trem2 knockout macrophages. *n* = 5 samples per group. **u**, **v**. Edu/DAPI staining images and quantification data of VSMCs co-cultured with IL-13-treated senescent Trem2 knockout macrophages. *n* = 5 samples/group. *p < 0.05, **p < 0.01, ***p < 0.001

To determine whether there is underlying crosstalk between macrophages with IL-13/Trem2 and VSMCs in vascular aging, we first tested the effects of aging macrophages treated with IL-13 on VSMC phenotypes in vitro. BMDMs were isolated from WT mice and induced to differentiate into macrophages. Then, the macrophages were treated with Ang II and IL-13, with PBS used as a control for the treatment with IL-13. The results of the western blotting analysis revealed a decrease in the expression of the marker proteins of the senescence phenotype (P21 and P53) in aging macrophages treated with IL-13 relative to the control (Fig. 5a, b). ELISA also revealed a decrease in the levels of the factors of the senescence-associated secretory phenotype (SASP), which included TNF-α, IL-1β, and IL-6, in aging macrophages treated with IL-13 (Fig. 5c). A coculture experimental system was subsequently established to examine the crosstalk between macrophages and VSMCs. This approach revealed that the IL-13-treated group suppressed the migration of these VSMCs (Fig. 5d, e), decreased the ROS levels, increased α-SMA expression (Fig. 5f-i), and inhibited the proliferation of these VSMCs (Fig. 5j, k). These findings suggested that IL-13 can delay the aging of macrophages and that IL-13-treated macrophages can attenuate the senescence phenotype of VSMCs in a paracrine manner. We further examined the IL-13 and Trem2 effects in senescent macrophages on VSMC phenotypes in vitro. We harvested BMDMs from WT or T2-KO mice. The results revealed that losing Trem2 expression in senescent macrophages not only reversed the expression changes of the senescence phenotype marker proteins and SASP factors caused by IL-13 treatment (Fig. 5l-n) but also reversed the changes in the above indicators of VSMC phenotypes caused by IL-13 treatment (Fig. 5o-v). The findings suggested that the combination of IL-13/Trem2 in macrophages enhances the ability of VSMCs to protect against vascular aging through paracrine signaling.

### IL-13/Trem2 combination promotes the transport of mitochondrial NAD^+^ via the Syk-Sp1-SLC25A51 pathway in aging macrophages

**Fig. 6:**
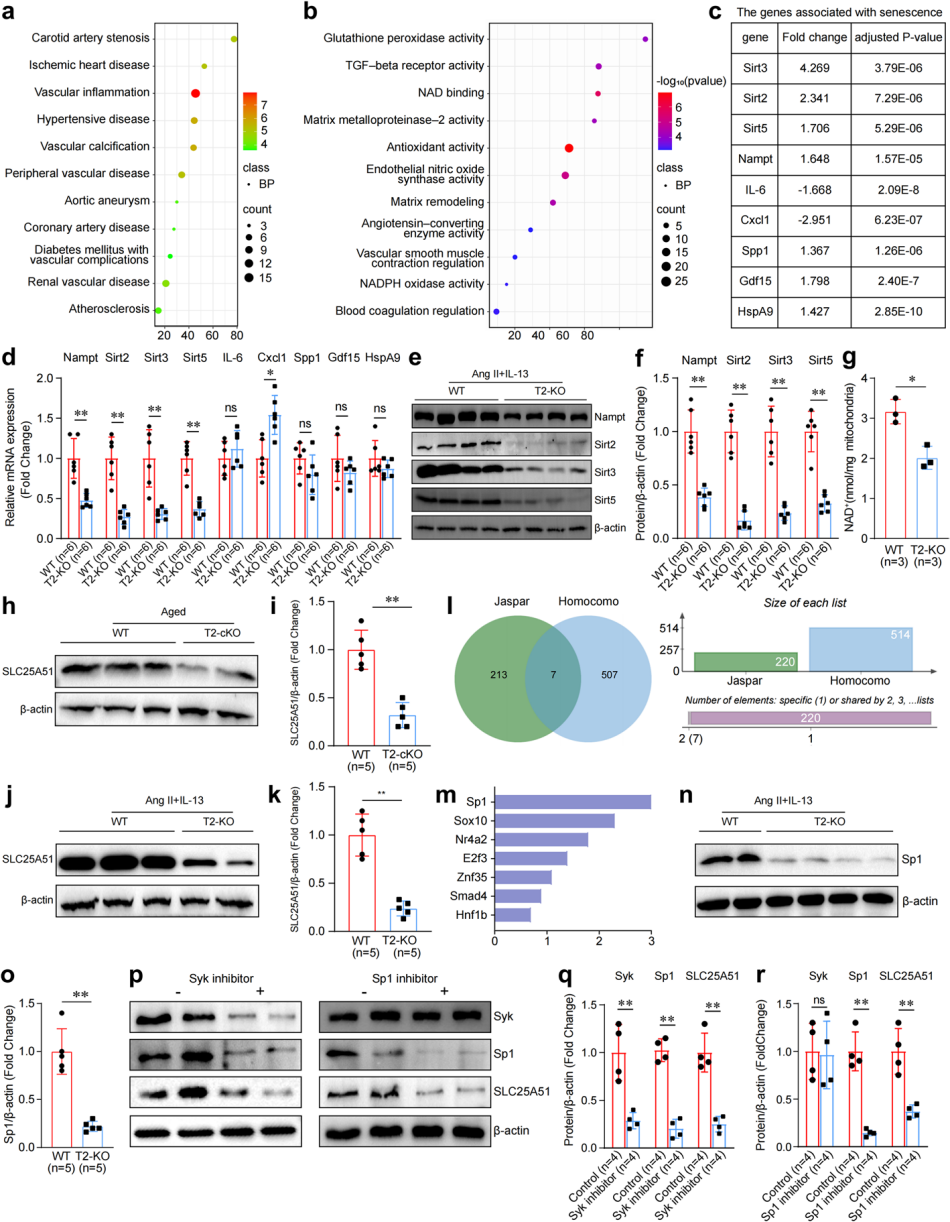
IL-13/Trem2 combination encourages the transport of mitochondrial NAD⁺ via Syk-Sp1-SLC25A51 pathway in aging macrophages. **a, b** RNA-seq analysis of the transcriptome of macrophages, which were derived from WT or T2-KO mice, with Ang II and IL-13. **c** Transcriptome profile data of the expression changes of senescence-related genes. **d** mRNA expression of senescence-related genes, including Nampt, Sirt2, Sirt3 and Sirt5 in IL-13-treated WT and T2-KO aging macrophages. *n* = 6 samples per group. **e, f** Western blotting images and quantification of senescence-related genes, including Nampt, Sirt2, Sirt3 and Sirt5 in IL-13-treated WT and T2-KO aging macrophages. *n* = 6 samples/group. **g** Measurement of the content of NAD^+^ in the mitochondria of IL-13-treated WT and T2-KO aging macrophages. *n* = 3 samples/group. **h, i** Western blotting and quantification of SLC25A51 in aortas samples of aged WT and aged T2-cKO mice. n = 5 samples/group. **j, k** Western blotting and quantification of SLC25A51 in WT and T2-KO aging macrophages treated with IL-13. *n* = 5 samples/group. **l, m** Schematic of the screening process for potential regulatory factors of SLC25A51 using the Jasper and Homocomo databases. **n, o** Western blotting images and quantification of Sp1 in IL-13-treated WT and T2-KO aging macrophages. *n* = 5 samples/group. **p-r** Western blotting images and quantification of Sky, Sp1 and SLC25A51 in ageing macrophages added with Syk or Sp1 inhibitors. *n* = 4 samples per group. *p < 0.05, **p < 0.01

To elucidate the mechanism regulated by the combination of IL-13 and Trem2 in aging macrophages, transcriptomic sequencing was performed following the treatment of macrophages derived from WT or T2-KO mice with Ang II and IL-13. Transcriptome examination determined that 745 genes were upregulated and 238 were downregulated in the T2-KO group in comparison to the WT group. Trem2 loss in aged macrophages treated with IL-13 enriched the genes participating in arterial diseases related to age, such as coronary artery disease and vascular calcification, indicating that the combination of macrophages and IL-13/Trem2 modulates a gene signature associated with age-dependent arterial pathologies (Fig. 6a). Functional clustering analysis revealed that several DEGs among them were associated with signaling pathways involved in senescence, vascular remodeling, and oxidative stress (Fig. 6b). Subsequent investigation of the overlapping DEGs among these top 20 pathways determined that the expression of several senescence-related genes, including Sirt3, Sirt2, Sirt5, Nampt, IL-6, Cxcl1, Spp1, Gdf5, and Hspa9, significantly changed in the T2-KO group (Fig. 6c). The western blotting and qPCR results confirmed that Trem2 deficiency downregulated Nampt, Sirt2, Sirt3, and Sirt5 expression in aging macrophages treated with IL-13 (Fig. 6d-f). As Sirts are a class of deacetylases dependent on NAD^+^ and Nampt is a crucial rate-limiting enzyme in NAD^+^ biosynthesis [23, 24], we hypothesized that Trem2 affects the phenotype of vascular aging by regulating the transport of mitochondrial NAD^+^. To test this hypothesis, we measured the mitochondrial NAD^+^ content and found that Trem2 knockout reduced the mitochondrial NAD^+^ content in aging macrophages treated with IL-13 (Fig. 6g), which indicated that the IL-13/Trem2 combination plays an anti-aging role in aging macrophages by regulating the transport of mitochondrial NAD^+^.

The recently identified mitochondrial NAD^+^ transporter known as SLC25A51 can transport cytoplasmic NAD^+^ into mitochondria, thus maintaining the stability of the NAD^+^ level in the mitochondria and facilitating the normal progression of various metabolic activities [25]. Next, the expression of SLC25A51 was determined in vivo and in vitro. The results revealed that SLC25A51 expression was lower in the aged aortas of the T2-cKO mice than in the WT mouse aortas (Fig. 6h, i). Subsequent western blotting also revealed that SLC25A51 expression was significantly downregulated in Trem2-/- aged macrophages treated with IL-13 (Fig. 6j, k), indicating that the IL-13/Trem2 combination regulated mitochondrial NAD^+^ transport via SLC25A51.

To identify the factors through which IL-13/Trem2 acts on SLC25A51, the Jasper database and the Homocomo database were searched; the assessment revealed that Sp1 might be the most crucial regulatory element for SLC25A51 (Fig. 6l, m). Western blotting analysis confirmed that Sp1 expression was significantly downregulated in Trem2-/- aged macrophages treated with IL-13 (Fig. 6n, o). Additionally, Syk, a downstream signal transducer of Trem2, has been reported to interact with the transcription factor Sp1 [12, 26]; we hypothesized that Trem2 enhances Sp1 expression by activating Syk in aging macrophages. The results revealed that the Sp1 and SLC25A51 expression levels declined significantly when the Syk inhibitor was added to aging macrophages, whereas only the SLC25A51 level was downregulated when the SP1 inhibitor was added to aging macrophages (Fig. 6p-r). These findings indicated that in aging macrophages, the combination of IL-13/Trem2 promotes the transport of mitochondrial NAD^+^ via the Syk-Sp1-SLC25A51 pathway.

### IL-13/Trem2 combination enhances beneficial crosstalk between macrophages and VSMCs through Syk-Sp1-SLC25A51 pathway-mediated upregulation of α-KG

**Fig. 7:**
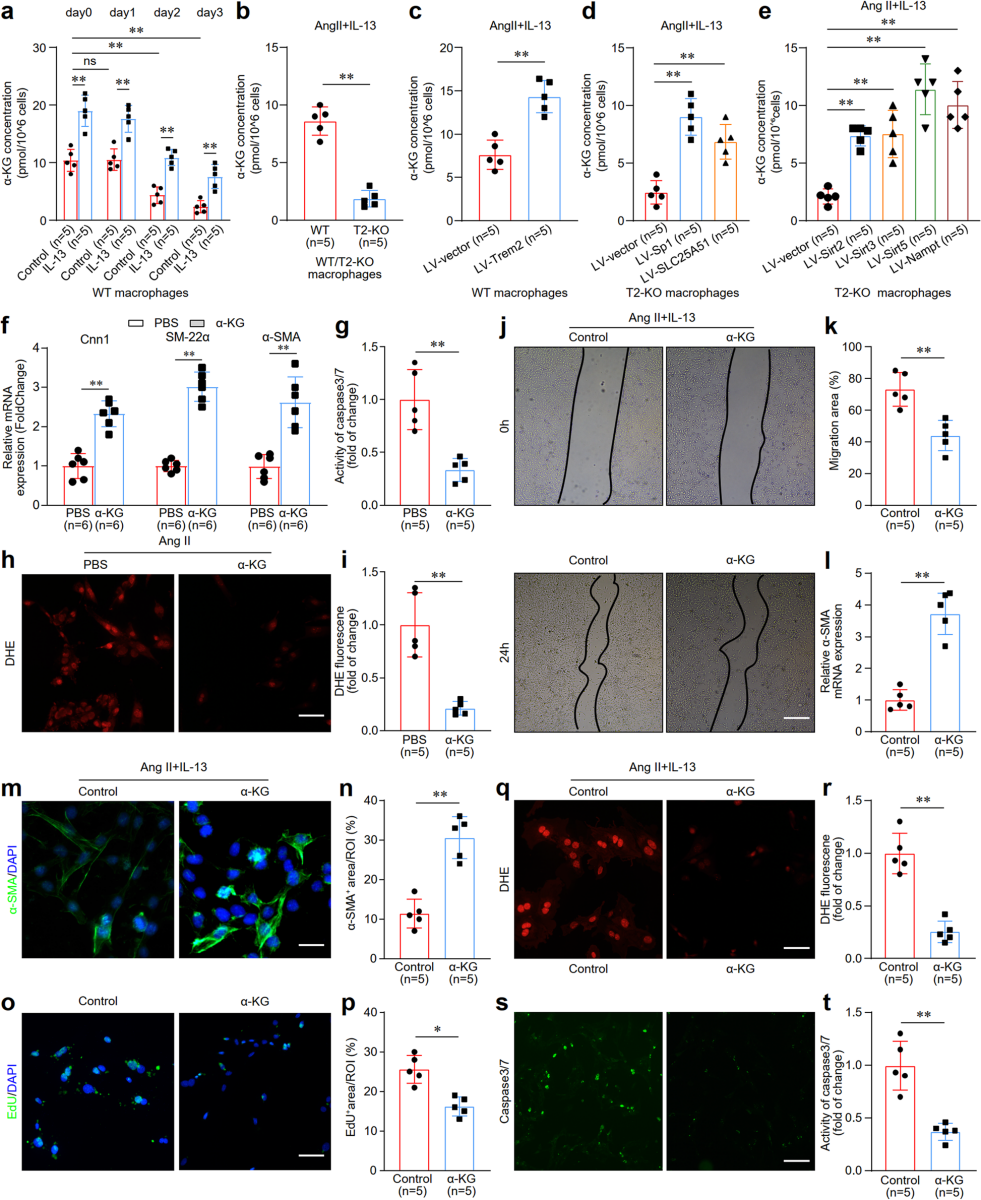
IL-13/Trem2 combination enhance the beneficial crosstalk between macrophage and VSMCs through Syk-Sp1-SLC25A51 pathway-mediated upregulation of α-KG. **a** Measuring the α-KG concentration in senescent macrophages upon Ang II stimulation at different time points with/without IL-13 treatment. *n* = 5 samples/group. **b** Measuring the α-KG concentration in IL-13-treated senescent macrophages isolated from WT and T2-KO mice. *n* = 5 samples per group. **c** Measuring the concentration of α-KG in senescent macrophages treated with IL-13 and then transfected with a Trem2-overexpressing lentivirus. *n* = 5 samples per group. **d** Measurement of the α-KG concentration in IL-13-treated T2-KO senescent macrophages transfected a lentivirus for Sp1 or SLC25A51 overexpression. *n* = 5 samples per group. **e** Measuring the concentration of α-KG in IL-13-treated T2-KO senescent macrophages transfected a lentivirus for Sirt2, Sirt3, Sirt5, or Nampt overexpression. *n* = 5 samples/group. **f** QPCR quantification of contractile protein markers Cnn1, SM-22α, and α-SMA in α-KG-treated senescent VSMCs. *n* = 6 samples/group. **g** Measurement of Caspase 3/7 activity in α-KG-treated senescent VSMCs. *n* = 5 samples per group. **h, i** DHE staining images and quantification in α-KG-treated senescent VSMCs. Scale bar: 100 µm.* n* = 5 samples per group. **j, k** Images and quantification of the wound-healing assay regarding the effects of α-KG on VSMC migration in a co-culture experimental system of IL-13-treated T2-KO senescent macrophages and VSMCs. Scale bar: 100 µm. *n* = 6 samples per group. **l** QPCR quantification of the effect of α-KG on contractile protein markers α-SMA in a co-culture experimental system of IL-13-treated T2-KO senescent macrophages and VSMCs. *n* = 5 samples per group. **m, n** Immunofluorescence staining and quantification of the effect of α-KG on contractile protein markers α-SMA in a co-culture experimental system of IL-13-treated T2-KO senescent macrophages and VSMCs. Scale bar: 100 µm. *n *= 5 samples per group. **o, p** Edu staining images and quantification regarding the effect of α-KG on cell proliferation of VSMCs in a co-culture experimental system of IL-13-treated T2-KO senescent macrophages and VSMCs. Scale bar: 100 µm. *n* = 5 samples per group. **q, r** DHE staining images and quantification data regarding the effect of α-KG on cell proliferation of VSMCs in a co-culture experimental system of IL-13-treated T2-KO senescent macrophages and VSMCs. Scale bar: 100 µm. *n* = 5 samples per group. **s, t** Caspase 3/7 activity images and quantification data regarding the effect of α-KG on cell apoptosis of VSMCs in a co-culture experimental system of IL-13-treated T2-KO senescent macrophages and VSMCs. Scale bar is 100 µm. *n* = 5 samples per group. *p < 0.05, **p < 0.01

As a key tricarboxylic acid cycle intermediate, α-KG metabolism depends on mitochondrial NAD^+^ levels and promotes a synthetic phenotype in VSMCs [27, 28]. We hypothesized that macrophage-derived α-KG via the IL-13/Trem2 interaction regulates VSMC phenotypes. In senescent macrophages, α-KG production decreased after 48 or 72 h of Ang II stimulation but was rescued by IL-13 treatment (Fig. 7a). We also found that Trem2 deficiency reduced the amount of α-KG in aging macrophages treated with IL-13 (Fig. 7b). In contrast, overexpression of Trem2 via a lentivirus increased the amount of α-KG in senescent macrophages treated with IL-13 (Fig. 7c). These results indicated that combining IL-13 and Trem2 enhances α-KG production to ameliorate macrophage senescence. In T2-KO senescent macrophages treated with IL-13, the overexpression of Sp1, SLC25A51, Sirt2, Sirt3, Sirt5, or Nampt using lentiviruses reversed the reduction in α-KG production caused by Trem2 deficiency (Fig. 7d, e vs. 7b). These findings indicated that the IL-13/Trem2 complex promotes α-KG production through the Syk-Sp1-SLC25A51 signaling pathway.

To test whether α-KG produced by the senescent macrophage line IL-13/Trem2 affects the VSMC phenotype, we first examined the effects of α-KG on the senescent VSMC phenotype. The results revealed that α-KG promoted the expression of the contractile proteins Cnn1, SM-22α, and α-SMA (Fig. 7f), inhibited cell apoptosis (Fig. 7g), and blocked oxidative stress in senescent VSMCs (Fig. 7h, i). These findings suggested that α-KG augments the transformation of senescent VSMCs into a contractile phenotype. Next, a coculture experimental system was established to examine the effects of α-KG produced by the senescent macrophages IL-13/Trem2 on the VSMC phenotype. The results showed that α-KG reversed the effects of promoting VSMC migration (Fig. 7j, k), proliferation (Fig. 7o, p), oxidative stress (Fig. 7q, r), and apoptosis (Fig. 7s, t) and reducing the expression of α-SMA (Fig. 7l-n) in Trem2 knockout senescent macrophages treated with IL-13. These findings indicated that α-KG serves as a mediator of the macrophage IL-13/Trem2 complex and VSMC phenotypes in vascular aging.

### Administration of α-KG counteracts the vascular aging and dysfunction triggered by macrophage Trem2 loss in vivo

**Fig. 8:**
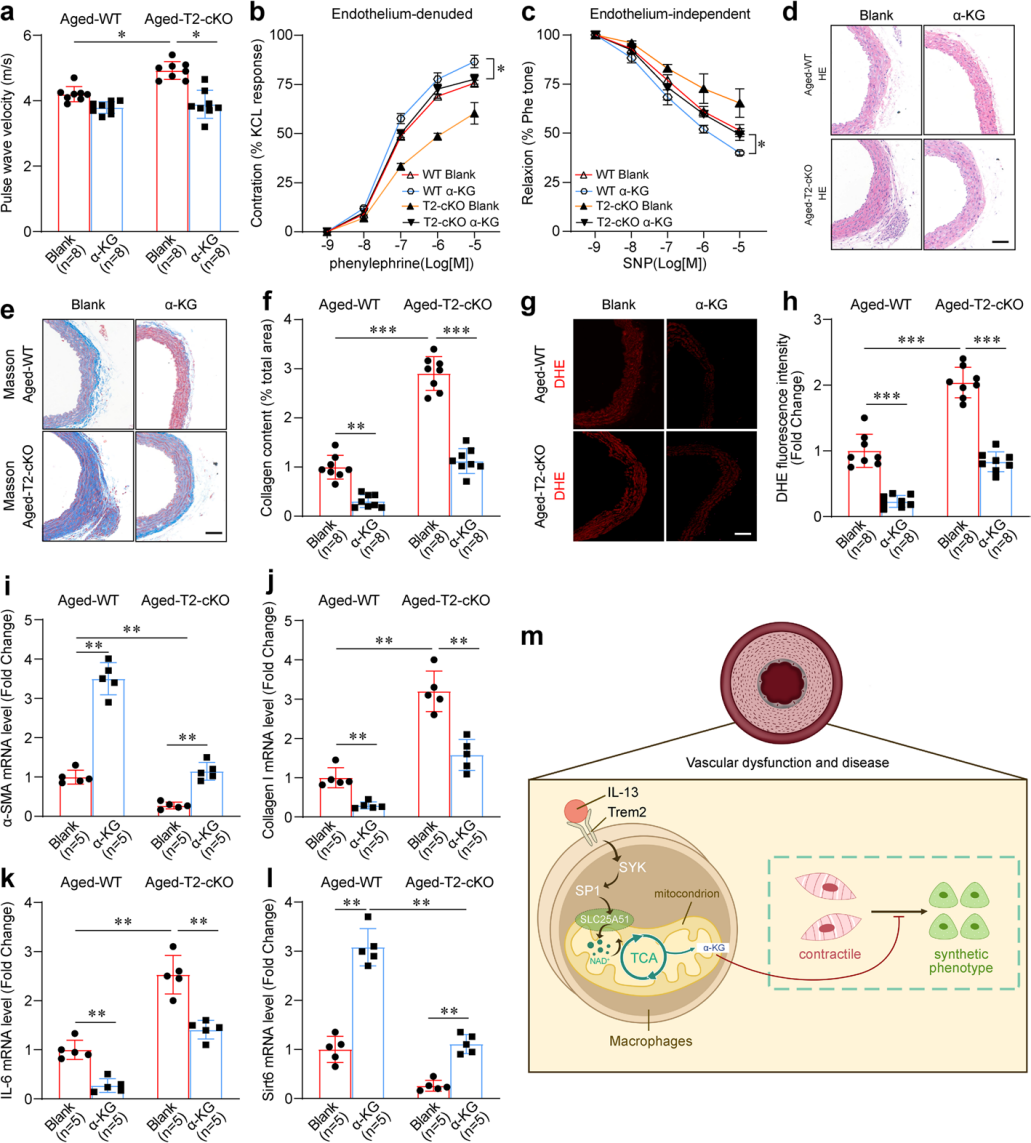
Administration of α-KG counteracts the vascular ageing and dysfunction triggered by macrophage Trem2 loss in vivo. **a** PWV measurements in four groups of aged mice. *n* = 8 samples/group. **b** Measurement of arterial vessel contractions mediated by PE in four groups of aged mice. *n* = 8 samples per group. **c** Measurement of arterial vessel relaxation mediated by SNP in four groups of aged mice. *n* = 8 samples per group. **d** H&E staining analysis on aortic samples from four groups of aged mice. Scale bar: 100 µm.* n* = 8 samples per group. **e, f** Masson staining images and quantification of aortic samples from aged mice. Scale bar: 100 µm. *n* = 8 samples per group. **g, h** DHE staining images and quantification on aortic samples from aged mice. Scale bar: 100 µm. *n* = 8 samples per group. **i-l** QPCR quantification of the expressions of Collagen I, α-SMA, IL-6 and Sirt6 in aortic samples from aged mice. *n* = 5 samples per group. **m** Working model: The macrophage IL-13/Trem2/Syk-Sp1-SLC25A51 axis mitigates vascular aging by enhancing mitochondrial NAD^+^ transport and α-ketoglutarate (α-KG) production. *p < 0.05, **p < 0.01, ***p < 0.001

To confirm the knockout efficiency of Trem2 in aged mice, we first performed Western blot analysis, which verified significantly reduced Trem2 expression in aged T2-cKO compared to WT mice (Fig. S3). Building on this validated model, we then investigated the effects of α-KG supplementation on vascular aging induced by macrophage-specific Trem2 deletion by comparing four groups of aged mice: WT without α-KG, T2-cKO without α-KG, WT with α-KG, and T2-cKO with α-KG. Compared to aged WT mice, aged T2-cKO mice presented significantly higher PWV values, but the increased PWV values were partly reversed after adding α-KG (Fig. 8a). Subsequent vascular constriction-relaxation tests revealed that vascular constriction induced by phenylephrine in aortas denuded of endothelium was reduced in aged T2-cKO mice and was partly reversed after α-KG supplementation, with similar results recorded for endothelium-independent relaxation (Fig. 8b, c). Other hallmarks of vascular aging, including aggravated thickening of the arterial media (Fig. 8d), increased collagen deposition (Fig. 8e, f), an enhanced oxidative stress response (Fig. 8g, h), could be reversed following α-KG supplementation in aged T2-cKO mice. Similar results were found for the expression of α-SMA, Collagen I, IL-6 and Sirt6 (Fig. 8i-l). These results revealed that α-KG administration alleviates the aging-induced vascular dysfunction triggered by macrophage Trem2 knockout. In addition, Fig. 8m illustrates the definitive mechanism through which the IL-13/Trem2 axis mitigates vascular aging, governed by α-KG-dependent metabolic crosstalk between macrophages and VSMCs.

## Discussion

Macrophages crucially influence vascular aging through their plastic responses to microenvironmental cues [5–7]. Trem2, a myeloid-specific immunoreceptor [12, 13], remains unexplored in vascular aging. We first demonstrated Trem2 upregulation in aged aortic macrophages. Its macrophage-specific deletion exacerbated vascular dysfunction in aged mice and reduced mitochondrial NAD^+^ transport enzymes, suggesting Trem2 regulates metabolic reprogramming in senescent macrophages.

Although the exact function of Trem2 in various diseases is not fully understood, several studies on Trem2 have focused on the CNS, demonstrating that the overexpression of Trem2 can suppress the inflammatory response in animal models of AD, multiple sclerosis, Parkinson’s disease, and other neurodegenerative diseases [12, 13, 29]. The expression and role of Trem2 in diseases related to age, such as cancer, diabetes, and atherosclerosis, have been reported recently, with contrasting effects [16–18, 30]. For example, Piollet et al. [31] demonstrated that knocking out Trem2 can accelerate necrotic core formation in atherosclerosis, whereas Patterson et al. [18] reported that Trem2 promoted lipid uptake by foamy macrophages and survival in atherosclerosis. These opposite results may be explained by Jay et al. [29] who reported that the loss of Trem2 reduces plaque number and area in the early stages of AD-related pathologies but increases plaque size and area in the late stages. Our findings provided novel information on vascular aging, revealing that Trem2 protein levels are elevated in aged aortas and that macrophage Trem2 deficiency exacerbates aging-induced vascular dysfunction and remodeling.

Vascular aging is characterized by various pathophysiological features, including oxidative stress, endothelial dysfunction, inflammation, thickening of the intima and media, and the progressive development of arterial stiffness [32, 33]. Consistent with these findings, the results of this study demonstrated that the loss of macrophage Trem2 in the aortas of aged mice triggered more severe inflammation and oxidative stress. This, in turn, led to worse arterial stiffness and impaired vascular constriction-relaxation function. Additionally, it increased the thickness of the arterial media, increased collagen deposition, and breakage of elastin fibers. These results suggested that macrophage Trem2 is crucial in mitigating degenerative vasculature changes related to age. Supporting our data, Trem2 deletion exacerbates neuroinflammation and neuronal apoptosis in AD models, whereas its overexpression confers protection [29, 34]. Liu et al. [35] further showed Trem2 upregulation reduces neuronal apoptosis and oxidative stress via PI3K–Akt signaling. We found that macrophage Trem2 loss impaired VSMC contraction, suggesting paracrine regulation of VSMC function in vascular aging. Further studies should delineate the specific downstream pathways and ligands through which macrophage Trem2 influences vascular aging.

Trem2 can detect damage to tissues and interact with an extensive range of ligands to execute functions that are diverse and dependent on context [12]. For example, in the brains of AD models, Trem2 can interact directly with pathological β-amyloid (Aβ) oligomers, as well as lipoproteins and apolipoproteins, to jointly inhibit plaque formation [36, 37]. In acute myeloid leukemia (AML), Xie et al. [38] reported that the binding of IL-34 to Trem2 can inactivate the extracellular signal-regulated protein kinase 1/2 signaling pathway, thus inhibiting the proliferation and differentiation of AML cells. However, the mechanism by which Trem2 deficiency affects vascular aging remains poorly understood. Our results indicated that in macrophages, the binding of IL-13 to Trem2 retards vascular aging by regulating the phenotype of VSMCs in a paracrine manner. Similarly, previous studies have shown that macrophages induced by IL-13 exhibit high efferocytosis and anti-inflammatory activities in the functional recovery of individuals with myocardial infarction and spinal cord injury, respectively [39, 40]. The results indicated that IL-13 is a ligand of Trem2 that protects against vascular aging.

We investigated how the interaction of IL-13 and Trem2 slows vascular aging at the molecular level. When Trem2 was knocked out, the mitochondrial NAD^+^ content in aging macrophages treated with IL-13 decreased. As reported in other studies [41, 42], intracellular NAD^+^ levels decrease during vascular aging, and restoring NAD^+^ homeostasis can reverse vascular senescence. We also elucidated the mechanism of IL-13/Trem2 interaction promotion of the transport of mitochondrial NAD^+^. SLC25A51 acts as a mitochondrial NAD^+^ transporter [25]. In immune cells, high-avidity ligand binding to DAP12–Trem2 fully activates DAP12, triggering downstream Syk signaling [43]. Consistent with these findings, when a Syk inhibitor was added to aging macrophages, the expression levels of Sp1 and SLC25A51 decreased significantly. In contrast, when an SP1 inhibitor was added to aging macrophages, only the level of SLC25A51 decreased. These results suggested that combining IL-13 and Trem2 promotes the transport of mitochondrial NAD^+^ through the Syk-Sp1-SLC25A51 pathway in aging macrophages. We also elucidated the intermediates involved in the crosstalk between VSMCs and macrophages mediated by the IL-13/Trem2 complex. Our findings revealed that α-KG acts as a key mediator, triggering phenotypic changes in VSMCs induced by the macrophage IL-13/Trem2 axis during vascular aging. Some studies have reported that α-KG has multiple functions, such as scavenging amino groups, suppressing oxygen radical generation, preventing lipid peroxidative damage, and inhibiting oxidative stress [44, 45]. Niemiec et al. [46] reported that dietary supplementation with α-KG can stabilize redox homeostasis and enhance arterial elasticity in aged mice. Consistent with these findings, our results indicated that α-KG supplementation effectively reversed vascular dysfunction induced by macrophage Trem2-knockout in aged mice. These findings suggested that both Trem2 and α-KG are potential treatment targets for combating diseases related to vascular aging.

This study has several limitations. First, using only male mice limits generalizability, given known sex differences in vascular aging [47]. Second, while the Syk-Sp1-SLC25A51 pathway partially mediates IL-13/Trem2 effects, transcriptome data suggest additional mechanisms are involved. Finally, although we focused on macrophage-to-VSMC signaling via α-KG, Trem2 may also act on endothelial cells and fibroblasts, requiring further investigation into intercellular crosstalk.

Our results revealed that Trem2 attenuated vascular aging by binding IL-13 to enhance mitochondrial NAD ^+^ transport and α-KG secretion. This α-KG-mediated macrophage-VSMC crosstalk identified Trem2 as a potential therapeutic target for vascular rejuvenation.

## Materials and methods

### Animals

Animals C57BL/6J wild-type (WT) and macrophage-specific Trem2 knockout (T2-cKO) mice were used in this study, as described previously [48]. To minimize potential confounding effects of sex hormones on vascular aging, only male mice were included in all experiments. All animals were housed under specific pathogen-free conditions with a 12-h/12-h light/dark cycle and provided with standard feed and water ad libitum. All animal procedures were approved by the Animal Ethics Committee of Shanghai Public Health Clinical Center and conducted in accordance with relevant ethical guidelines. Genotyping protocols for Trem2-cKO and Lyz2-cre mice are provided in Fig.S4. To investigate the role of Trem2 in vascular aging, age-matched male WT and T2-cKO littermates were maintained until designated timepoints of 2 months (young) and 24 months (aged), with a sample size of 13–15 mice per group. For angiotensin II (Ang II)-induced vascular aging modeling, 8–10-week-old C57BL/6J male mice were randomly assigned to receive continuous subcutaneous infusion of either Ang II (1.3 mg/kg/day; Sigma-Aldrich, A9525) or an equal volume of saline control for 28 days via osmotic minipumps, as adapted from an established protocol [47]. To evaluate the efficacy of α-ketoglutarate (α-KG) supplementation in modulating vascular aging in vivo, 18-month-old male WT and T2-cKO mice were randomly allocated into four experimental groups and provided free access to either standard chow or chow supplemented with 2% α-KG (MedChemExpress, 328–50–7) for six months.

### Transcriptome sequencing and qPCR analysis

Total RNA was extracted from mouse aortic tissues and AngII-stimulated macrophages (72 h) using TRIzol or RNAiso Plus reagents. RNA quality was verified by spectrophotometry (Nanodrop). For transcriptome analysis, libraries were prepared with NEBNext Ultra II RNA Kits and sequenced on an Illumina HiSeq X10 platform. Raw data were processed with DeSeq2 against the GRCh38 genome, followed by enrichment analysis of differentially expressed genes (DEGs).

For qPCR, RNA was reverse-transcribed to cDNA and amplified with SYBR Green Master Mix (Thermo Fisher Scientific). Primer sequences are listed in Table S1. Gene expression levels were quantified by the 2–ΔΔCt method with β-actin as the endogenous control.

### Western blotting

Samples were lysed in RIPA buffer supplemented with phosphatase and protease inhibitors. Protein concentrations were determined using a BCA assay kit, and equal amounts of lysates were separated by 10% or 12.5% SDS-PAGE and transferred to PVDF membranes (Bio-Rad). After blocking with 5% skim milk in TBST, membranes were incubated overnight at 4 °C with primary antibodies (detailed in Table S1), followed by incubation with HRP-conjugated secondary antibodies for 1 h at room temperature. Protein bands were visualized by enhanced chemiluminescence and quantified by densitometry.

### Histological and immunostaining analyses

Aortic tissues from young and aged mice were fixed in 10% paraformaldehyde, paraffin-embedded, and sectioned at 5–15 µm thickness. Histological staining ((hematoxylin and eosin (H&E), Masson's trichrome, and elastic van Gieson (EVG)) was performed as previously described [49]. For immunohistochemistry/immunofluorescence, sections underwent antigen retrieval, endogenous peroxidase blockade, and blocking, followed by incubation with primary antibodies (Trem2, NLRP3, MMP-9, α-SMA) at 4 °C overnight and species-matched secondary antibodies at 37 °C for 30 min (fluorescent-conjugated for α-SMA). All slides except α-SMA samples were developed with DAB and counterstained with hematoxylin. After dehydration and mounting, images were acquired using a digital slide scanner (3DHISTECH Pannoramic MIDI II) or fluorescence microscope (Nikon), and quantitatively analyzed by blinded investigators with ImageJ.

### Measurement of PWV

Pulse wave velocity (PWV) was determined using a vascular function analyzer that was noninvasive. First, the mice were anesthetized, followed by catheterization of both the carotid artery and the femoral artery. Next, the spatial separation between the two measurement sites was determined, and the temporal lag between the pulse wave arrivals at these two points was recorded. PWV was computed as the quotient of the distance and the time difference.

### Measurement of arterial constriction-relaxation function

The mice were killed by cervical dislocation, and the thoracic aorta was promptly excised via thoracotomy. After removing perivascular fat and connective tissues in Krebs–Henseleit (KH) solution, the aorta was sliced into 2–3 mm thick arterial rings. The aortic rings were positioned in a myograph chamber and equilibrated in KH solution. The following steps were subsequently performed: phenylephrine (PE, 10^–9^–10^–5^ μmol/L) was used for aortic contraction, acetylcholine (Ach, 10^–9^–10^–5^ μmol/L) was used for relaxation that was endothelium-dependent, and sodium nitroprusside (SNP, 10^–10^–10^–5^ μmol/L) was used for relaxation that was endothelium-independent. The changes in isometric tension were recorded in detail throughout the procedure.

### Bone marrow-derived macrophage culture

Bone marrow-derived macrophages (BMDMs) isolated and cultured in this study, as described previously [48]. Briefly, bone marrow cells were harvested from murine femurs and tibias and differentiated for 7 days in complete Dulbecco's Modified Eagle Medium (DMEM) supplemented with 10 ng/mL M-CSF. The resulting BMDMs were stimulated with Ang II to model the vascular aging microenvironment.

### Murine VSMC culture, proliferation, and migration

Mouse aortic smooth muscle cells (ATCC) were maintained in DMEM with 10% fetal bovine serum (FBS) and 1% penicillin–streptomycin (PS). Cell proliferation was evaluated using an EdU kit (Sangon Biotech, E607204) per manufacturer's protocol.

For migration assessment, Transwell assays used serum-free DMEM suspensions (1 × 10⁶ cells/mL) in 8-μm inserts with 10% FBS as chemoattractant. Wound-healing assays were performed by scratching confluent monolayers and measuring closure after 24 h.

α-SMA immunofluorescence was conducted on coverslip-seeded VSMCs fixed with 4% PFA, then incubated with primary and fluorescent secondary antibodies.

### BMDM and VSMC coculture assays

Initially, BMDMs were stimulated with Ang II (1 mmol/L) for 72 h. Subsequently, the supernatants were harvested and filtered through a 0.45-µm membrane filter (Millipore-Sigma). Then, the VSMCs were cultured in the obtained conditioned media for 24–48 h.

### Flow cytometry analysis

Aortic tissues were processed into single-cell suspensions through collagenase I/elastase digestion and filtered through 70 μm strainers. After red blood cell lysis and dead cell exclusion, cells were stained with an extended antibody panel including CD45 (BV510), CD11b (FITC), F4/80 (PE-Cy7), Ly6G (APC-Cy7), Ly6C (PE), Trem2 (APC), CD31 (BV421), CD146 (PE/Dazzle™ 594), CD90.2 (BV650), PDPN (BV711), and α-SMA (PE-Cy5.5). Cell populations were identified through sequential gating: live CD45⁺ immune cells (macrophages: CD11b^+^ F4/80^+^ Ly6G^−^; neutrophils: CD11b^+^ Ly6G^+^) and live CD45^−^ stromal cells (endothelial cells: CD31^+^ CD146^+^; fibroblasts: CD31^−^ CD90.2^+^ PDPN^+^; VSMCs: CD31^−^ α-SMA^+^). Trem2 expression was specifically analyzed across all populations to confirm macrophage-restricted upregulation in aged aortas.

### ELISA

Levels of macrophage-derived TNF-α, IL-1β, and IL-6 were measured using commercial ELISA kits (Abcam) according to the manufacturer's instructions. Absorbance at 450 nm was recorded with a microplate reader, and protein concentrations were determined based on a standard curve.

### Reactive oxygen species and apoptosis detection

As described in our previous study [50], reactive oxygen species (ROS) levels were measured in aortic tissues and cultured cells using dihydroethidium (DHE) and DCFH-DA staining, respectively, with fluorescence intensity quantified by fluorescence microscopy. Apoptosis was assessed by TUNEL assay in aortic tissues and by Caspase 3/7 assay kit in cells.

### Immunoprecipitation and glutathione S-transferase pull-down assays

To conduct the assay for immunoprecipitation (IP), lysates from HEK-293T and RAW 264.7 cells were incubated overnight with anti-IL-13 or anti-Trem2 antibodies at 4 °C, followed by protein A/G Plus-agarose incubation for 2 h. Beads were washed, boiled in SDS buffer, and immunoprecipitated proteins were analyzed by SDS-PAGE and Western blotting.

In the glutathione S-transferase (GST) pull-down assay, GST beads were used to immobilize GST-IL-13 fusion proteins, followed by incubation with a binding buffer containing proteins tagged with His (His-Trem2, His-Trem2 [NT-aa1-132], and His-Trem2 [NT-aa133-227]). The expression of the GST fusion proteins was SDS-PAGE-confirmed, followed by staining with Coomassie Brilliant Blue, and the GST-IL-13–His-Trem2 interaction was verified through western blotting.

### Colocalization of Trem2 and IL-13 in senescent macrophages

Macrophages grown on coverslips were stimulated with Ang II for 72 h to induce senescence. After fixation with 4% paraformaldehyde and permeabilization with 0.1% Triton X-100, cells were blocked with 1% BSA and incubated with specific primary antibodies, followed by fluorescence-conjugated secondary antibodies and DAPI. Colocalization of Trem2 (green) and IL-13 (red) was analyzed by fluorescence microscopy.

### Detection of the mitochondrial NAD^+^ content

Initially, we isolated BMDMs from WT and Trem2 knockout mice. Mitochondria were subsequently extracted from these cells using the Qproteome Mitochondria Isolation Kit (Qiagen). To determine the NAD^+^ content in the mitochondria, the NAD/NADH Assay Kit (Abcam, ab65348) was used, and the measurement was conducted strictly following the manufacturer’s instructions.

### LC–MS/MS analysis

A stable Trem2-overexpressing macrophage line was established for LC–MS/MS analysis [48]. Briefly, Trem2-overexpressing macrophages were stimulated with Ang II for 72 h, followed by immunoprecipitation of 1 × 10⁷ cells using Millipore's protocol (17–500). Coomassie blue-stained protein bands from 4–12% SDS-PAGE were excised, digested with trypsin at 37 °C for 18 h, and the resulting peptides were desalted and concentrated for LC–MS/MS analysis.

### Lentiviruses

The full-length sequences of Trem2, Sp1, SLC25A51, Sirt2, Sirt3, Sirt5, and Nampt were individually ligated to pLKO.1-puro-CMV-TurboGFP and then transformed into *E. coli* cells. After verifying the vectors, the vectors were cotransfected with a packaging mixture into FT293 cells to generate gene-specific lentiviruses, and the lentiviral infection titers were measured.

### Statistical analysis

All statistical analyses were performed using the GraphPad Prism 9 software. Appropriate statistical tests were selected according to the characteristics of the data. Normally distributed data with homogeneous variances underwent an unpaired two-tailed Student’s t-test for two-group comparisons, whereas one-way ANOVA, then Dunnett’s test was conducted for multiple-group comparisons. two-way ANOVA was used to further assess the effect of α-KG supplementation on vascular function over time. All results were deemed to have statistical significance at *P* < 0.05 (two-sided). Significance levels were denoted by asterisks: *p < 0.05 and **p < 0.01, ***p <0.001 and “NS” denoted a nonsignificant difference.

## Supplementary Information

Supplementary Material 1.

## Data Availability

The raw data of this study can be obtained by contacting the corresponding authors via email. For the Jasper database and HOCOMOCO database involved in Figs. 6L and M, the access links are as follows: JASPAR Database: https://jaspar.elixir.no/ HOCOMOCO Database: https://hocomoco13.autosome.org/H13CORE
