# Supplementary material for: Macrophage Trem2 deficiency aggravates aging-induced vascular remodeling by acting as a non-classical receptor of interleukin-13
Source: Mol Biomed. 2025 Dec 29;6:153. doi: 10.1186/s43556-025-00377-1 (PMC12748396; doi:10.1186/s43556-025-00377-1)
Supplement: Supplementary file 1 — Supplementary Material 1. [file 43556_2025_377_MOESM1_ESM.docx]

**Supplementary Information**

**Macrophage Trem2 deficiency aggravates aging-induced vascular remodeling by acting as a non-classical receptor of interleukin-13**

Youming Chen^1#^, Zhaoxiang Zeng^2#^, Zetao Wei^3#^, Yi Zhan^4^, Luling Wu^5^*,·Xinlin Zhu^6,7^*, Meifang Li^8,9^*

^1^ Department of Infectious Diseases and Immunology, Shanghai Public Health Clinical Center, Fudan University, Shanghai 201508, China

^2^ Department of Vascular Surgery, Shanghai General Hospital, Shanghai Jiaotong University, Shanghai 201620, China.

^3^ Department of Emergency, Dan Zhou People’s Hospital, Danzhou, Hainan 271706, China

^4^ Department of Radiology, Shanghai Public Health Clinical Center, Fudan University, Shanghai 201508, China

^5^ Department of Endoscopy, Shanghai Pulmonary Hospital, School of Medicine, Tongji University, Shanghai 200082, China

^6^ Department of Dermatology, Shanghai Key Laboratory of Medical Mycology, Shanghai Changzheng Hospital, Naval Medical University, Shanghai 200003, China

^7^ The Center for Fungal Infectious Diseases Basic Research and Innovation of Medicine and Pharmacy, Ministry of Education, Shanghai 200433, China

^8^ Department of Emergency, Shanghai Sixth People's Hospital Affiliated to Shanghai Jiao Tong University School of Medicine, Shanghai 200233, China

^9^ Department of Emergency, Jinjiang Municipal Hospital (Shanghai Sixth People's Hospital Fujian), Jinjiang, Fujian 362200, China.

**Keywords** vascular aging, Trem2, macrophage, IL-13, metabolic reprogramming, α-KG

* Corresponding authors:

Luling Wu

Email: [wululing_1120@126.com](mailto:wululing_1120@126.com)

Xinlin Zhu

Email: [zhuxinlin1994@163.com](mailto:zhuxinlin1994@163.com)

Meifang Li

Email: [15821955054@126.com](mailto:15821955054@126.com)

^#^ Youming Chen, Zhaoxiang Zeng and Zetao Wei contribute equally to this work.

**Fig. S1**


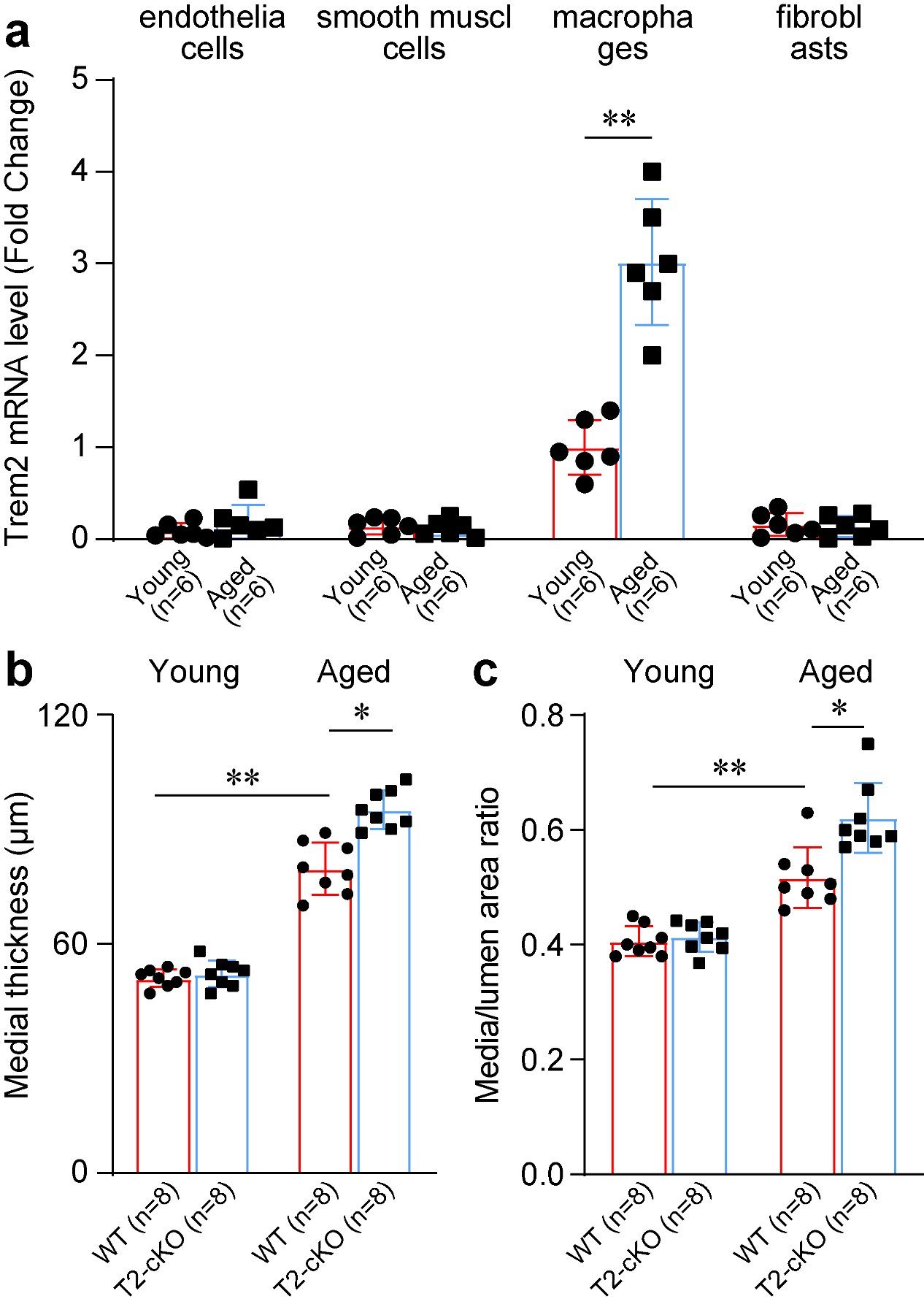


**Fig. S2**


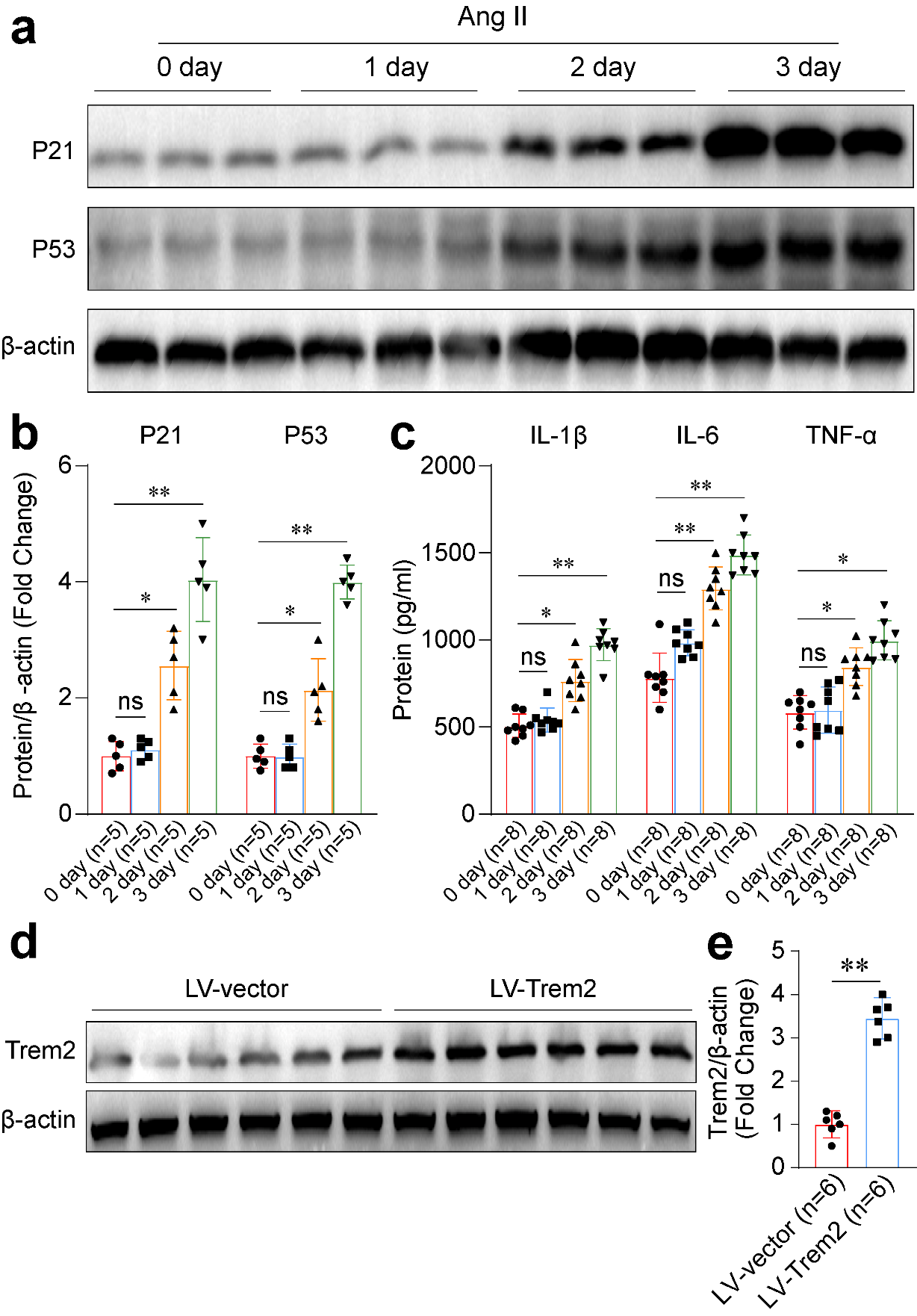


**Fig. S3**


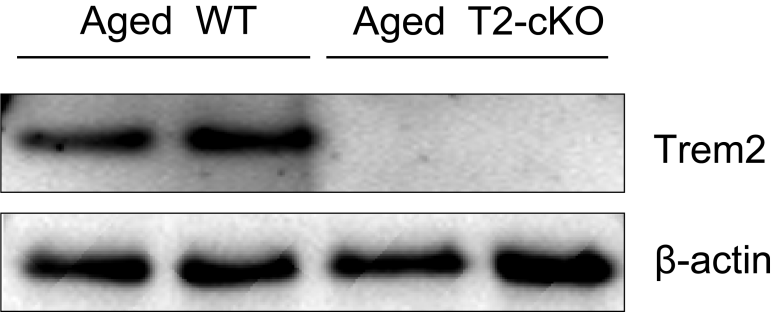


**Fig. S4**


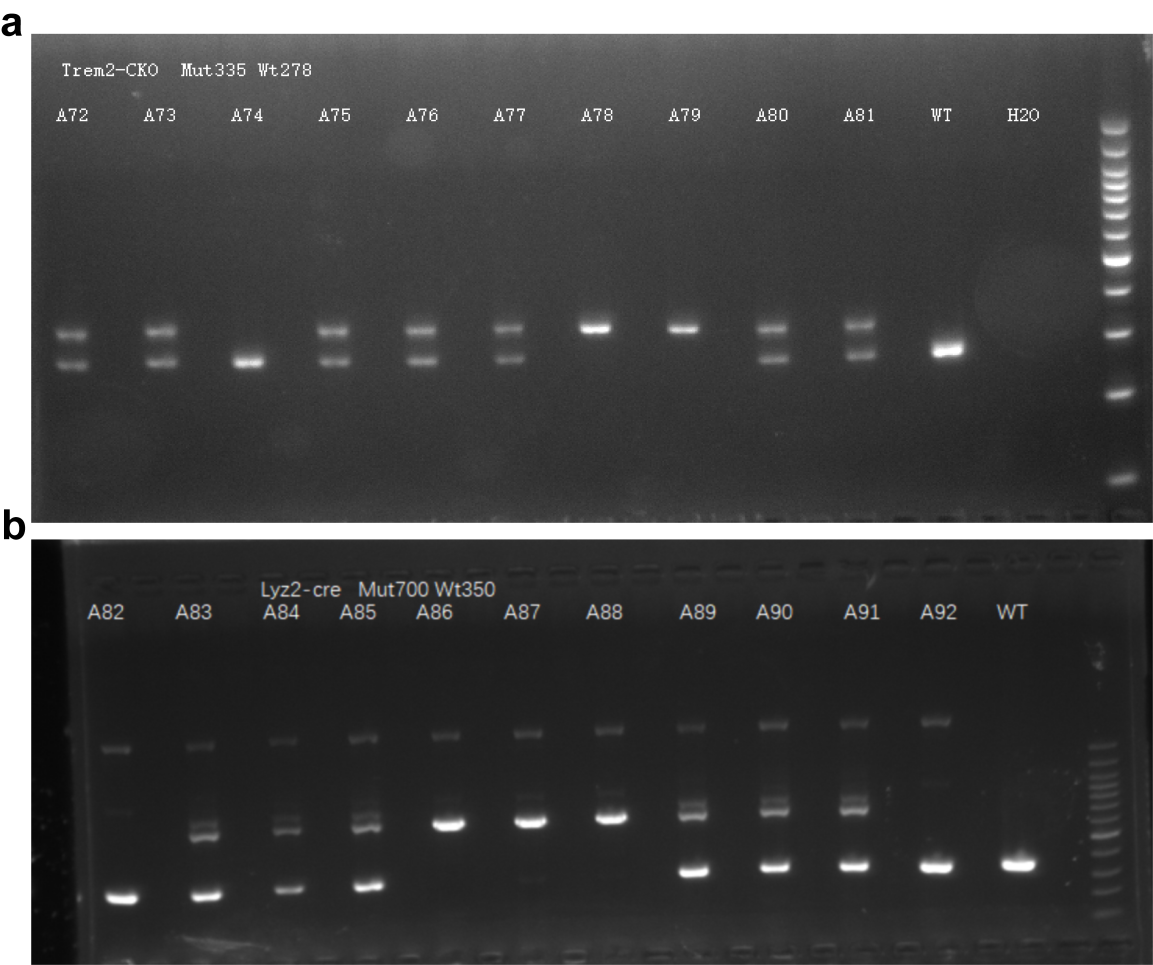


**Table S1. Primer sequences** **for qPCR and antibodies used in western blotting analysis of this study**

| 1. **Primer sequences for qPCR** | | |
| --- | --- | --- |
| **Gene** | **Forward Primer** | **Reverse Primer** |
| Trem2 | CTGGAACCGTCACCATCACTC | CGAAACTCGATGACTCCTCGG |
| Mmp9 | GCAGAGGCATACTTGTACCG | TGATGTTATGATGGTCCCACTTG |
| α-SMA | CCCAGACATCAGGGAGTAATGG | TCTATCGGATACTTCAGCGTCA |
| SM-22α | GCTATGGCATTAACACCACGG | CCCAGGTTCATTAGTGTCCGC |
| Collagen I | GCTCCTCTTAGGGGCCACT | ATTGGGGACCCTTAGGCCAT |
| Collagen III | CTGTAACATGGAAACTGGGGAAA | CCATAGCTGAACTGAAAACCACC |
| Mmp2 | ACCTGAACACTTTCTATGGCTG | CTTCCGCATGGTCTCGATG |
| IL6 | CTGCAAGAGACTTCCATCCAG | AGTGGTATAGACAGGTCTGTTGG |
| Nlrp3 | ATTACCCGCCCGAGAAAGG | CATGAGTGTGGCTAGATCCAAG |
| Cxcl1 | ACTGCACCCAAACCGAAGTC | TGGGGACACCTTTTAGCATCTT |
| Ccl2 | TAAAAACCTGGATCGGAACCAAA | GCATTAGCTTCAGATTTACGGGT |
| Tnf-α | CAGGCGGTGCCTATGTCTC | CGATCACCCCGAAGTTCAGTAG |
| Cxcl2 | CCAACCACCAGGCTACAGG | GCGTCACACTCAAGCTCTG |
| Nampt | GCAGAAGCCGAGTTCAACATC | TTTTCACGGCATTCAAAGTAGGA |
| Sirt2 | GCGGGTATCCCTGACTTCC | CGTGTCTATGTTCTGCGTGTAG |
| Sirt3 | GAGCGGCCTCTACAGCAAC | GGAAGTAGTGAGTGACATTGGG |
| Sirt5 | CCAGTTGTGTTGTAGACGAAAGC | TTCCGAAAGTCTGCCATATTTGA |
| Spp1 | ATCTCACCATTCGGATGAGTCT | TGTAGGGACGATTGGAGTGAAA |
| Gdf15 | CTGGCAATGCCTGAACAACG | GGTCGGGACTTGGTTCTGAG |
| Hspa9 | ATGGCTGGAATGGCCTTAGC | GCACCCTTGATTGCTTCTGATG |
| 1. **Antibodies used for western blotting analysis** | | |
| **Primary antibodies** | **category numbers** | **suppliers** |
| anti-Trem2 | #70765 | Cell Signaling Technology |
| anti-IL-13 | ab79277 | Abcam |
| anti-Sirt3 | ab264041 | Abcam |
| anti-Sirt2 | ab211033 | Abcam |
| anti-Sirt5 | ab259967 | Abcam |
| anti-SLC25A51 | ab237054 | Abcam |
| anti-SP1 | ab227383 | Abcam |
| anti-Syk | #2712 | Cell Signaling Technology |
| anti-β-actin | #4967 | Cell Signaling Technology |
| anti-P53 | ab26 | Abcam |
| anti-Nampt | ab236874 | Abcam |

**Figure legends**

**Fig. S1.** Quantification of the cells, the thickness of the middle artery and the middle artery area-to-vascular lumen ratio in aorta samples of young and old WT mice. **a** QPCR analysis of Trem2 levels in different cells from aortas samples of young and old WT mice via flow sorting. n = 6 samples per group. **b** Middle artery thickness quantification in aorta samples of young and aged mice. n = 8 samples per group. **c** Quantification of the middle artery area-to-vascular lumen ratio in aorta samples of young and aged mice. n = 8 samples/group.

**Fig. S2.** Ang II can be utilized to simulate aging phenotype in macrophages. **a, b** Western blotting images and quantification of the levels of aging markers P21 and P53 levels in macrophages at day 0, 1, 2, 3 after Ang-II stimulation. n = 5 samples/group. **c** ELISA detection of the SASP levels, including IL-1β, IL-6 and TNF-α on macrophages at day 0, 1, 2, 3 after Ang-II stimulation. n = 8 samples per group. **d, e** Western blotting images and quantification of the Trem2 overexpression efficiency in macrophages using lentivirus. n = **6** samples/group.

**Fig. S3.** Assessment of Trem2 Knockout in Aged Mice by Western Blotting. The efficiency of Trem2 knockout in aged mice was assessed by Western blot analysis.

**Fig. S4.** Trem2-cKO and Lyz2-cre mice genotyping. **a** Trem2-cKO mouse genotyping. WT: One band at 278 bp; heterozygous: two bands at 335 and 278 bp; homozygous: one band at 335 bp. **b** Lyz2-cre mouse genotyping. WT: One band at 350 bp; heterozygous: two bands at 350 and 700 bp; homozygous: one band at 700 bp.

**Table S1. Primer sequences** for qPCR and antibodies used in western blotting analysis of this study.
